# Integrating Immune Checkpoint Blockade with Anti-Neo/Mutated Antigens Reactivity to Increase the Clinical Outcome of Immunotherapy

**DOI:** 10.3390/vaccines3020420

**Published:** 2015-05-21

**Authors:** Giorgio Parmiani, Cristina Maccalli, Michele Maio

**Affiliations:** 1 Italian Network for Bio-therapy of Tumors-(NIBIT)-Laboratory, c/o University Hospital of Siena, V. le Bracci, 16, Siena 53100, Italy; E-Mail: crimaccalli@gmail.com (C.M.); 2 Division of Medical Oncology and Immunotherapy, University Hospital of Siena, V. le Bracci, 16, Siena 53100, Italy; E-Mail: mmaiocro@gmail.com (M.M.)

**Keywords:** immune checkpoint antibodies, neo/mutated tumor antigens, melanoma immunotherapy

## Abstract

Antibodies to immune checkpoints have entered the clinical arena and have been shown to provide a clinical benefit for metastatic melanoma and, possibly, for other tumors as well. In this review paper we summarize this therapeutic activity and underline the functional mechanisms that may be involved. Among them, we discuss the so far neglected role of tumor-associated antigens (TAAs) deriving from tumor somatic mutations and summarize the results of recent trials showing the immunogenic strength of such TAAs which can be specifically targeted by T cells activated by immune checkpoint antibodies. Finally we discuss new immunotherapy approaches that involve the combination of self/shared- or neo-TAAs-based vaccines and immune checkpoint blockade antibodies, to increase the clinical response of metastatic melanoma patients.

## 1. Introduction

### Immune Checkpoint Blockade Agents in Human Solid Tumors

In the last few years several new immune checkpoint blockade agents targeting either the Cytotoxic T-Lymphocyte Antigen 4 (CTLA-4) or the Programmed Death 1 (PD-1)/Programmed Death Ligand 1 (PD-L1) signaling, have been developed and some of them already approved for clinical use by regulatory agencies in Europe and/or USA (e.g., Ipilimumab, Nivolumab, Pembrolizumab). The mechanism of immune checkpoint antibodies is still an area of research but may involve the activation of TAA-specific T effector cells [1] and promoting their *in vivo* survival [2] and migration to tumors [3] with no need to down-regulate T regulatory cells. This process is even more strongly induced when combinations of different antibodies are used (e.g., ipilimumab combined with anti-PD-1 or anti-PD-L1; [4,5,6]) as documented in murine systems [7] and appears to involve the release of IL-2 and proliferation of T lymphocytes within the tumor microenvironment (MEV) [8].

Since tumor MEV is immunosuppressive, it is not yet clear how checkpoint antibodies can overcome such an immune suppression. However, even in animal models, as shown in the early studies by Allison’s group [9,10], it was not established the quantity and quality of TAAs targeted in mice in which regressions of the transplantable B16 melanoma or sarcomas after CTLA-4 blockade were observed. The CTLA-4^–/–^ gene-deleted mice developed autoimmunity against different tissues, thus one could argue that many self/differentiation TAAs can be targeted by checkpoint blockade. The variety of clinical trials in which the anti-melanoma activity of immune checkpoint antibodies was demonstrated [11,12,13,14,15], however, showed that a good proportion of patients were only partially susceptible to this treatment with a subpopulation of them being even completely resistant to checkpoint blockade antibodies. Since recent studies showed that T lymphocytes reactive against somatically mutated neo-TAAs could be isolated from patients clinically responding to ipilimumab, we propose that the variability of the clinical response at the single patient level may depend on the frequency and the immunogenicity of his TAA repertoire (see below).

We also suggest that the expression of a large number of neo/mutated TAAs could be relevant even in the context of different immunotherapy approaches such as peptide antigen-based vaccination that can mimic the anti-tumor T-cell activation, and immune checkpoint antibodies, that can potentiate the previously induced immune responses.

This may occur either with immune checkpoint antibody monotherapy (e.g., Ipilimumab, Nivolumab) [5,15,16] or with combined therapies with two different checkpoint antibodies [17] and perhaps even by combining Ipilimumab with chemotherapy [18].

## 2. TAAs in the Immunotherapy of Cancer: Are We Selecting the Right Immunogenic Target?

Human TAAs include epitopes recognized by T cells in the context of class I or class II major histocompatibility complex (MHC) molecules. TAAs have been grouped according to their molecular characterization and tissue distribution. The group of shared/self-differentiation and cancer/testis TAAs has been used in the last two decades as therapeutic vaccines in patients with different forms of cancer. The initial phase I and II trials have been conducted with a single or two peptide epitopes while during the last few years, HLA class I- and/or II-restricted multiple peptides have been administered simultaneously in an attempt to avoid immune selection of TAA-negative tumor cells by tumor reactive T lymphocytes (Immuno-editing) [19]. Peptide-based vaccines have been usually given emulsified in the IFA-like adjuvant Montanide ISI 51 or pulsed onto autologous dendritic cells (DCs) and/or admixed with different cytokines [20]. These phase I-II trials showed variable frequencies (20%–60%) of patients developing an anti-vaccine T cell response, while tumor regression has been reported in a minority of cases (10%–20%).

An attempt to vaccinate patients with autologous tumor-derived gp96 heat shock proteins (known to bind mutation-derived neo-TAAs) [21] led to tumor-specific T cell immune response in 50%–60% of metastatic colon carcinoma patients [22] with evidence of better survival in immune responders as compared to non-responders.

In any events, the results of many clinical trials of immunotherapy based on self/weak/differentiation TAAs were altogether quite disappointing, even when combined with immune checkpoint agents [10]. Moreover, one of the first studies of the addition of CTLA-4 antibody failed to increase the clinical response to a Granulocyte/Macrophage/Colony Stimulating/Factor (GM-CSF) gene-transduced tumor cell vaccine (GVAX) while augmenting the tumor destruction by patient T cells [9].

However, a set of strongly immunogenic tumor antigens is represented by TAAs deriving from non-synonimous somatic mutations of genes, either genes driving tumor development (CANgenes) or with other functions, whose massive characterization has been precluded until recently. Now the development of cost-effective, high-throughput DNA sequencing platforms allows the rapid identification of all the somatic mutations included in a cancer cell genome [23,24]. This method, combined with a bioinformatics analysis for T cell epitope prediction and established reverse immunology approaches provides an integrated strategy to identify patient-specific unique TAAs in a relatively short time compatible with their potential use in the clinic [25,26,27,28,29,30]. Using this approach, Snyder *et al.* [31] recently reported that the mutated TAAs of human melanoma cells can generate strong epitopes that were important for the Ipilimumab activating immune reaction in melanoma patients and correlating with clinical responses to the therapy. Similar results were obtained in NSCLC patients given a PD-1 antibody [32] and in chemically induced mouse sarcomas treated with anti-CTLA-4 and/or anti-PD-1 antibodies [33]. The long term neglected role of neo- (mutated) TAAs which constitute the foundation of the modern tumor immunology [34] was due to the technological complexity for their molecular characterization. 

This new genomic approach allows the identification of a TAAs landscape that was thought to be characteristic of the ipilimumab therapy [31]. Thus, as suggested also by the results of Gubin *et al.* [33], we propose that a reaction against neo-TAAs can occur even with other immunotherapy approaches, including the peptide-based vaccines [27]. Of note, the clinical responses to immune check-point agents have been observed in histologically and biologically dissimilar neoplasms. Though in some cases shared gene mutations have been found (e.g., mutated BRAF gene shared between melanoma (50%) and breast cancer (5%–10%)) in these cancer types, it is feasible that effective immune responses directed against patient’s specific mutated antigens were activated by immunomodulating antibodies. The number of clinically useful mutations, however, can vary substantially among tumors [35] and even from different lesions of the same tumor with skin melanoma bearing a high frequency of mutated TAAs (0.5–100 mutations/megabase). This is in line with melanoma etiology that may depend on strong mutagenic agents (e.g., UVB). This can explain the high susceptibility of human melanoma to immune checkpoint blockade since, besides their systemic activity, these antibodies can migrate to tumor lesions and then activate T cells within the tumor thus counteracting the immunosuppression of the MEV. Such a phenomenon may induce the highest neo-antigens T cell responses and conferring a better survival to patients bearing high frequency of somatically mutated TAAs [27,35]. Tumors with variable mutation load showed responsiveness to immune checkpoint blockade agents, since these molecules can activate/potentiate highly reactive immune responses to neoantigens. The effectiveness of these immune responses can be independent on the number of mutations of tumors while may be affected by the antigenic potency and the expression by tumor tissues of neo-antigens.

## 3. Can Cancer Stem Cells Represent Target for Immunotherapy?

A potential *novel target* of immunotherapy is represented by *cancer stem/initiating cells* (CSCs) whose destruction may interrupt the growth and diffusion of the tumor. Thus CSCs could represent a crucial target of immunotherapy in case they express strong and specific TAAs. To elucidate this aspect and to determine whether such an hypothesis can be tested, we have evaluated the antigenic profile of glioblastoma and colorectal cancer CSCs [36,37]. These CSCs revealed an impaired expression of crucial immunological molecules like class I and II HLA as compared with non-CSC counterparts; susceptibility to T cell-mediated cytotoxicity was also reduced. Several studies have addressed the reasons of the limited clinical outcome of vaccination against CSCs [38,39]. In fact, we found that CSCs from colorectal cancer express tumor-specific neo/mutated TAAs [29] which, however, can hardly be recognized by the immune system owing to the immunosuppressive factors released by CSCs and their low expression of MHC antigens [39].

In addition to the previously defined immune-escape mechanisms (e.g., down-regulation of HLA/peptide complexes by tumor cells, release of immunosuppressive molecules), it has recently been shown that new factors may prevent tumor rejection even in the presence of an ongoing tumor-specific immune reaction induced by the vaccine. One example is represented by the up-regulated IL-4 signaling associated with colorectal CSCs [37]. Moreover, the activation of T regulatory lymphocytes and of myeloid-derived suppressor cells (MDSCs) are also relevant. We have found MDSC both in the blood and tumor tissue of patients with metastatic melanoma and colorectal cancer [40]. These principles have now been incorporated in designing new vaccination protocols with multi peptides in melanoma and early prostate cancer patients [41].

## 4. Combination of Different Immunotherapy Agents

Combining checkpoint blockade and TAAs T cell response in human solid tumors resulted, however, in conflicting observations (see Table 1).

**Table 1:** Recent trials of combination of immune checkpoint inhibitors and vaccines in human tumors.

Vaccine	Tumor	N. Patients	Clinical Response	Immune Response	Adj.	mAb	Reference
MART-1, Gp100, Tyros	M Melanoma	75	Improved *vs.* historical controls	>MART-1-specific T cells	IIFA	Anti-CTLA-4	[ 42]
NY-ESO-1 + MART-1	M Melanoma	90	25% RR	NY-ESO-1+ MART-1	IFA	anti-PD-1	[ 43]
PROSTVAC	CRPC	30	58% ^#^	>T cells >NK cells	ND	Anti-CTLA-4	[ 44]
Gp100	Melanoma	676	OS = 10 *vs.* 6.4 mos	N. D.	IFA	Anti-CTLA-4	[ 11]
GVAX	Melanoma, Ovarian carcinoma	9	Me (3/3) necrosis. Ovarian cancer >SD	5/7 patients T cell response	No	Anti-CTLA-4	[ 45]
GVAX	CRPC	28	>OS	PBDC	No	Anti-CTLA-4	[ 46]
PSA-TRICOM	CRPC	30	58%	>T cells to MUC1 or PSA peptides	No	Anti-CTLA-4	[ 47]

Adj.: adjuvant; mAb: immune checkpoint blockade monoclonal antibody; N.D. = not done; ^#^: evaluated by PSA levels; OS = overall response. CRPC: Castration Resistant Prostate Cancer; IFA = Incomplete Freunds’ adjuvants; GVAX = Granulocyte, Macrophage colony stimulating Factorreleasing tumor cells; PBDCs = Peripheral blood DCs.

In fact, combination of ipilimumab with GVAX increased the T cell response and clinical response in mice with melanoma [9], but these results were not duplicated in other studies combining vaccination and ipilimumab [11]. The combination of vaccination with gp100 plus ipilimumab did not resulted in improved clinical responses as compared to ipilimumab alone. The optimization of the combination of immune checkpoint agents plus vaccination need to be further explored. The choice of the TAAs to be used for these combinations is still an open question. Clinical studies based on anti-PD-1 treatment for patient’s refractory to previous ipilimumab therapy have been performed and are still ongoing [43]. Questions are still open regarding the optimal dose and schedule of administering immune checkpoint blockade agents and their sequential treatments and the identification of patients who can benefit from these treatments.

## 5. Conclusions

The recent studies on the mechanisms of anti-tumor activity of immune checkpoints antibodies indicate that the patients’ immune system is involved in the therapeutic effect of these biological agents and that the expression of a specific subgroup of TAAs, namely the neo/mutated ones, appear to be relevant in eliciting a clinically beneficial reaction in terms of T cell-mediated destruction of tumor cells. Thus, the genomic analysis (as next generation sequencing) of which neo/mutated TAAs are expressed by the individual tumor appears to be a pre-requisite for the efficacy of this type of treatment. It is also relevant that histologically and biologically different tumors [35] can share a molecular target, thus facilitating the use of the same antibody for different neoplasms and reducing the present high cost of this treatment.

*Literature review sections.* Literature dealing with the relationship between neo-mutated antigens and immune checkpoint antibody clinical activity was reviewed through PubMed/MEDLINE.
